# Modifiable Risk Factors for Dementia Among Migrants, Refugees and Asylum Seekers in Australia: A Systematic Review

**DOI:** 10.1007/s10903-022-01445-2

**Published:** 2023-01-18

**Authors:** Mohammad Shoaib Hamrah, Larissa Bartlett, Sunny Jang, Eddy Roccati, James C. Vickers

**Affiliations:** Wicking Dementia Research and Education Centre, University of Tasmania, Private Bag 143, Hobart, TAS 7001 Australia

**Keywords:** Migrants, Refugees, Asylum seekers, Dementia, Systematic review

## Abstract

While the prevalence of non-communicable disease risk factors is understood to be higher among migrants than for people born in host nations, little is known about the dementia risk profile of migrants, refugees and asylum seekers. This systematic review examines published literature to understand what is currently reported about 12 identified modifiable risk factors for dementia among migrants, refugees, and asylum seekers residing in Australia. Three literature databases (PubMed/CINAHL/MEDLINE) were systematically searched to find articles reporting excessive alcohol consumption, traumatic brain injury, air pollution, lack of education, hypertension, hearing impairment, smoking, obesity, depression, physical inactivity, diabetes, and limited social contact in Australia’s migrant, refugee and asylum seeker population samples. Papers were systematically reviewed following PRISMA guidelines. A total of 763 studies were found, of which 676 articles were excluded, and 79 articles remained. Despite wide variability in study design, size and purpose, the prevalence and correlates of modifiable risk factors of dementia appears markedly different among the studied samples. Compared with Australian-born participants, migrant samples had a higher prevalence of depression, social isolation, physical inactivity and diabetes mellitus. Insufficient information or conflicting evidence prevented inference about prevalence and correlates for the remaining dementia risk factors. A better understanding of the prevalence and correlates of modifiable dementia risk factors is needed in Australia’s migrant, refugee and asylum seeker populations. This information, together with a deeper understanding of the contextual and cultural contributing factors affecting people who arrive in Australia through differing pathways is needed before preventive interventions can be realistically targeted and sensitively implemented.

**Supplementary Information:**

The online version contains supplementary material available at 10.1007/s10903-022-01445-2.

## Introduction

The number of international migrants has risen above 280 million globally, reflecting a continued rise across all regions of the world [1]. Nearly half of all international migrants were born in Asia, primarily from India, China and other countries in South Asia [2]. In 2020, it was estimated there were 281 million international migrants in the world, equal to 3.6% of the global population [3].

Voluntary migration can be acomplicated process, sometimes taking several years and involving significant exposure to stressors throughout pre‐migration, transit and resettlement [4, 5]. Referred to as a ‘healthy migrant effect’, migrants tend to be healthier than native residents, especially during the first 5–10 years post- immigration. New migrants bring healthier habits and lifestyles [6, 7], yet long-term health risks seem to be exacerbated for refugees and migrants [8] possibly due to limited access to timely medical services for disease prevention, treatment, and care [9]. For example, rates of reported chronic health conditions, such as cardiovascular disease and diabetes are higher in migrant samples than the populations in which they resettle [10]. This is also true for dementia, where research indicates the prevalence is significantly higher within migrant groups than host populations [11].

Australia is home to the highest number of migrants in Oceania, almost 30% of the total national population [12]. Australia recognises the poor health of migrants and refugees as a pressing issue in terms of clinical, societal, healthcare and service provision [8, 13]. Within this context, a focus on supporting healthy ageing and cognitive functioning is important, given the high prevalence of Australians with a migrant background [14], and statistics showing dementia remains underdiagnosed among migrant populations [15].

Dementia is now recognised as the leading cause of disability and dependency among older adults, carrying an estimated global cost estimate of US$1 trillion [16]. Amid failing pharmaceutical trials and emerging evidence for the beneficial effect of modifiable risk factors, a strong focus on public health initiatives that can achieve dementia risk reduction has emerged [17]. The 2020 report of the Lancet Commission on Dementia Prevention, Intervention and Care [18] identified 12 behavioural and lifestyle-related risk factors that account for 40% of the risk of developing dementia. For example, low educational attainment in early life (< 45 years) and hypertension, obesity, hearing loss, traumatic brain injury (TBI), and alcohol misuse or abuse during midlife [45–65 years] are understood to exacerbate risk. Risk factors that are present in later life (age > 65 years) are smoking, depression, physical inactivity, social isolation, diabetes, and air pollution [18]. Many of these dementia risk factors cluster around inequalities, in particular in vulnerable populations [18]. Targeting these risk factors requires positive health promotion messaging, but also ground level societal action in order to improve the circumstances of vulnerable people, enabling them to manage their own long-term health.

Dementia incidence is rising in low-income and middle-income countries [18], a source of the majority of migrant populations around the world [2]. Modifiable risk factors are more prevalent in Black, Asian and minority ethnic groups who are known to be socially disadvantaged [18]. Migrant vulnerability has also been reported in the UK, where the prevalence of dementia was higher among African-Caribbean migrants compared to UK-born people [19]. Little is known about the dementia risk profile of migrants and refugees in an Australian context, however, rates of hypertension, high blood cholesterol, physical inactivity, smoking, obesity/overweight and diabetes appear to be higher in Australian migrants and refugees than non-immigrant Australians [20–22]. Given these health conditions are known to exacerbate dementia risk, Australian migrants may be at a higher risk of dementia than their non-immigrant Australian counterparts.

Public health efforts to prevent future dementia incidence through risk reduction first require an understanding of risk profiles within target populations. Understanding dementia risk profiles in migrant communities supports the World Health Organization’s (WHO) global action plan to improve the health of refugees and migrants for 2019–2023 [23]. This aligns with initiatives from around the world seeking to facilitate service access for migrant communities, as accessing health services can be challenging for international migrants due to language and cultural barriers, discrimination, financial burden, and unfamiliar administrative processes and health systems [23].

Developing an evidence base about modifiable dementia risk factors among migrants, refugees and asylum seekers will provide insights into key areas of public health research, practice and resource planning. This will provide support for targeting interventions in at-risk groups, as well as identifying knowledge gaps for risk factor prevalence. Further, this work is crucial for designing and implementing appropriate primary prevention efforts aiming to reduce future dementia incidence in Australia’s culturally and linguistically diverse (CALD) populations.

There is currently no cohesive picture of modifiable dementia risk factors in Australian migrant populations. Australia is home to a large number of migrants, and this paper offers the first step toward understanding their adherence to dementia risk factors, by providing a systematic review of published research literature that reports on the 12 identified modifiable dementia risk factors in Australia’s migrant, refugee and asylum seeker populations.

## Methods

This protocol was registered in PROSPERO (CRD42021267347) and the Preferred Reporting Items for Systematic reviews and Meta-Analyses (PRISMA) guidelines were followed [24]. Please refer to supplementary materials for the PRISMA checklist (Table S1).

### Search Strategy

Three databases (PubMed/CINAHL/MEDLINE) were systematically searched for articles published between January 2000 and December 2020. Hand-searching of relevant review articles was also undertaken to identify additional primary studies. Controlled search terms from the MeSH thesaurus were applied with Boolean commands as follows: (refugee OR migrant OR immigrant OR asylum seeker AND (depression OR smoking OR education OR hearing loss OR traumatic brain injury OR hypertension OR alcohol OR obesity OR social isolation OR physical inactivity OR air pollution OR diabetes) AND (Australia) NOT (USA OR UK) (Table S2).

### Selection of Studies

Studies were included if they: (1) reported original research (no reviews); namely, longitudinal, case control, prospective cohort, retrospective cohort, cross-sectional, and case series; (2) contained multiple subjects; (3) included human subjects only; (4) reported results for one or more of 12 risk factors for dementia [18]; (5) the study sample included immigrants, migrants, refugees or asylum seekers; (6) with participants aged 50 years and over; and (7) resident in Australia. Studies were excluded if they: (1) did not provide sufficient information for the purposes of this review; (2) were conference abstracts, editorials, review or theoretical articles and books. After duplicates were removed the list of potentially included articles was screened for inclusion by two authors (MSH and LB), first by title/abstract, then by full text. Both authors (MSH and LB) screened title, abstracts and full texts. If MSH and LB disagreed, one of the other authors (EH or SJ) provided a final decision. Information extracted from each study included the author and date of publication, study design, study population, sample size, location, outcomes of interest, and key findings.

### Quality Assessment of the Included Studies

The quality of the included studies was reviewed against the National Institutes of Health [25] criteria by the first author, MSH. Scores were provided for each study against 14 items in the NIH Quality Assessment Tool for Controlled Intervention Studies (Table S3). or the Quality Assessment Tools for Observational Cohort and Cross-sectional Studies (Table S4).

### Data Synthesis

A narrative synthesis was performed to understand what has been published in the academic literature about modifiable dementia risk factors in migrant, refugee and asylum seeker populations residing in Australia. The risk factors included were depression, smoking, education attainment, hearing loss, traumatic brain injury (TBI), hypertension, excessive alcohol consumption, obesity, social isolation, physical inactivity, diabetes and air pollution [18].

## Results

### Description of the Included Studies

**Fig. 1:**
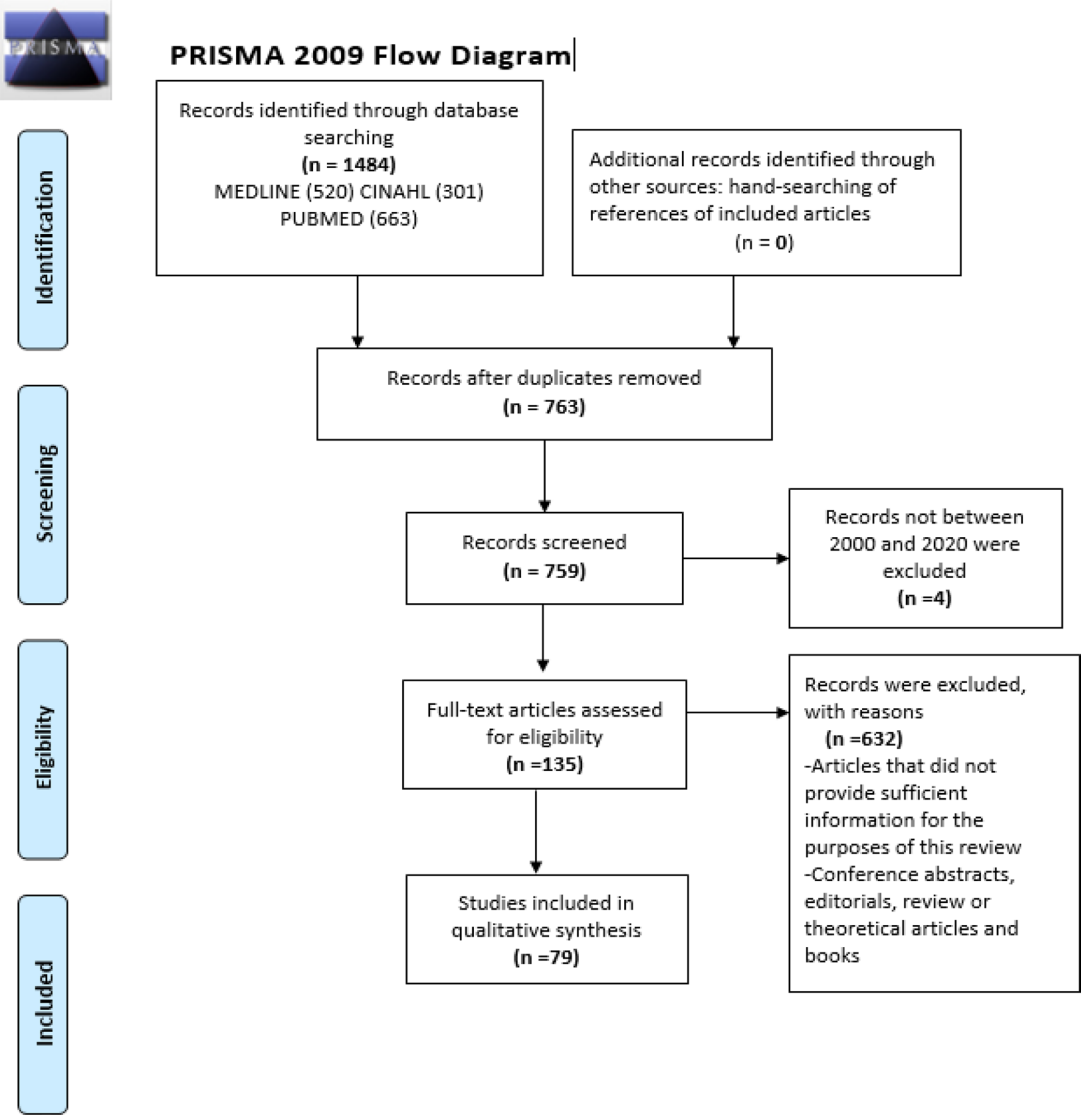
Preferred Reporting Items for Systematic Review and Meta-Analyses (PRISMA) diagram for systematic review of dementia risk factors in migrants, refugees and asylum seekers in Australia

**Fig. 2:**
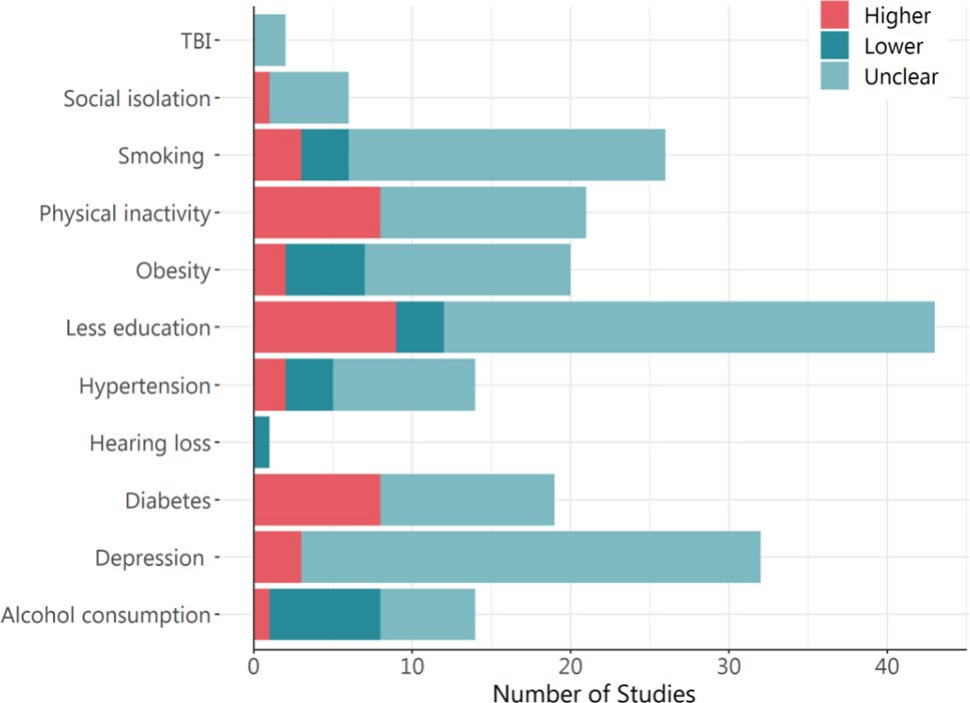
Number of studies identified in systematic review reporting on Lancet Commission’s 12 modifiable risk factors

The initial search returned 763 studies, of which 676 articles were excluded, and 79 articles selected according to the inclusion criteria (Fig. 1). Several of the included papers reported data collected from large research cohorts. Table 1 presents the full list of included studies. Some of the risk factors were more frequently studied in target samples (e.g. depression, diabetes) than others (e.g. hearing loss, social isolation). No studies reported on air pollution. The findings from our systematic review are reported below for each of the 12 modifiable dementia risk factors. Summary results are presented in Table 2 and illustrated in Fig. 2.

**Table 1:** Study characteristics of included studies

#	Author	Study design	Study name (if provided)	Sample origin	Outcomes of interest	Sample size
1	Almeida et al. (2015) [26]	Longitudinal study, follow-up period 6 years	Health In Men Study (HIMS)	Northern Europe, Mediterranean, others	Smoking, alcohol use, education, depression, physical activity	5276
2	Astell-Burt et al.( 2013) [27]	Longitudinal study, follow-up period 3 years	45 and Up Study	Scottish, Welsh, Irish, Danish, French, Swiss, German, Dutch, Spanish, Italian, Greek, Polish, Maltese, Lebanese, Croatian, Indian, and Chinese	Obesity, physical activity	214,807
3	Bilal et al. (2021) [28]	Cross-sectional study		Saharan African	Depression, diabetes	170
4	Brock et al. [29]	Cross-sectional study		Vietnam	Smoking, alcohol use, physical inactivity	225
5	Caperchione et al. (2011) [30]	Cross-sectional study		Bosnian, Arabic speaking, Filipino and Sudanese communities	Physical inactivity	110
6	Cerin et al. (2019) [31]	Cross-sectional study		China	Physical inactivity	91
7	Chen et al. (2019) [32]	Longitudinal study, follow-up period 2.4 years	Mental health of humanitarian migrants (HMs)	Participants were born in 35 different countries	Loneliness, social isolation, education	1723
8	Chen et al. (2017) [33]	Longitudinal study, follow-up period 6 months	Building a New Life in Australia Survey (BNLA)	Participants were born in 35 different countries	Loneliness, education, social isolation	2399
9	Choi et al. (2012) [34]	Randomised controlled trial (RCT)	Internet delivered Cognitive Behaviour Therapy (CBT) study	China, Hong Kong, Taiwan, Australia, Vietnam, Malaysia and America	Education, depression	109
10	Chou (2007) [35]	Longitudinal study, follow-up period 1 year	Longitudinal Survey of Immigrants to Australia (LSIA)	Asian countries, Western and developed countries and other countries	Smoking	431
11	Christopoulou and Lillard (2015) [36]	Longitudinal study, follow-up period 6 months	Household, Income and Labour Dynamics in Australia (HILDA) survey	British	Smoking	7063
12	Dassanayake et al. (2011) [37]	Cross-sectional	Data were drawn from the Australian National Health Survey (2001)	New Zealand, Oceania, UK & Ireland, North-Western Europe, Southern & Eastern Europe, North Africa, Middle East, Americas, South East Asia, and others	Physical inactivity	4956
13	Drummond et al. ( 2011) [38]	Cross-sectional		Liberia or Sierra Leone	Obesity, education, depression	51
14	El Masri et al. (2019) [39]	Longitudinal study, follow-up period 4 years	45 and Up Study	Participants were born in 35 different countries	Hypertension, diabetes, physical inactivity, smoking	41,940
15	Feng et al. (2014) [40]	Longitudinal study, follow-up period 3 years	45 and Up Study	Participants were born in 22 different countries	Smoking, physical inactivity, alcohol use	217,498
16	Gallegos et al. (2019) [41]	Longitudinal study, follow-up period 32 months	The Living Well Multicultural—Lifestyle Modification Program	Afghani, Arabic‐speaking, Burmese, Pacific and South Sea Islander, Sri Lankan, Sudanese and Vietnamese	Hypertension, education, physical inactivity	700
17	Gholizadeh et al. (2009) [42]	Cross-sectional		Women of Turkish, Iranian, and Persian backgrounds	Smoking, physical inactivity, depression, education	55
18	Goh et al.(2010) [43]	Cross-sectional		China	Depression, education	58
19	Guo et al.(2015) [44]	Longitudinal study, follow-up period 3 years	45 and Up Study	Australia, Northeast Asia, Southeast Asia, Europe	Obesity, smoking, education, obesity, physical inactivity, hypertension, alcohol	263,356
20	Hamrah et al. (2020) [45]	Cross-sectional		Afghanistan	Education, depression, isolation, loneliness	66
21	Hauck et al. (2011) [46]	Longitudinal study, follow-up period 3 years	Victorian Population Health Survey (VPHS)	East Europeans, South Europeans, North-West Europeans, East Asians, South Asians, Middle Eastern, Pacific	obesity, education	15,783
22	Hodge et al. (2004) [47]	Longitudinal study, the average length of follow-up was 4.4 years in Greek and Italian migrants and 4.0 years in the Australian- born participants	Melbourne Collaborative Cohort Study (MCCS)	Italy, Greece, Australia	Diabetes, smoking education alcohol and obesity	34,097
23	Ibiebele et al.(2000) [48]	Cross-sectional		India, Sri Lanka, Pakistan, Bangladesh and Burma	Diabetes, obesity	552
24	Jarallah and Baxter (2019) [49]	Longitudinal study, follow-up period 6 months	BNLA Wave 1	The Middle East and North Africa including Iran and Iraq, Central & Southern Asia, Afghanistan and others	Education, loneliness	2399
25	Jatrana et al. (2014) [50]	Longitudinal study, follow-up period 6 years	Labour Dynamics in Australia HILDA study	East Europeans, South Europeans, North-West Europeans, East Asians, South Asians, Middle Eastern, Pacific	Smoking, education, physical inactivity	6321
26	Jiang et al. (2017) [51]	Cross-sectional		China	Smoking, education	382
27	Jin et al. (2017) [52]	Longitudinal study, follow-up period 3 years	45-and-Up Study	China	Obesity, smoking, physical inactivity, hypertension	3220
28	Jin et al. (2017) [52]	Longitudinal study, follow-up period 3 years	45-and-Up Study	China	Obesity, smoking, physical inactivity, hypertension	266,696
29	Joshi et al. (2018) [53]	Longitudinal study, follow-up period 11 years	HILDA	The United Kingdom, United States of America, New Zealand, Canada, Ireland and South Africa, Italy, Germany, Vietnam, the Philippines, the Netherlands, China and India	Smoking, education	12,634
30	Kang et al. (2020) [54]	Longitudinal study, follow-up period 5.9 years	45-and-Up Study	Southeast Asia, southern and eastern Europe, southern and central Asia, Northeast Asia, northern and western Europe (excluding the United Kingdom and Ireland), sub-Saharan Africa, and the Middle East, Oceania (excluding New Zealand), Australia, the Americas (excluding North America)	Smoking, diabetes, hypertension, depression, obesity, education	115,988
31	Kartal et al. (2019) [55]	Cross-sectional study		Bosnia	Education, depression	138
32	Kiropoulos et al.(2011) [56]	RCT	The effects of Multicultural Information on Depression Online	Italy, Greece	Depression, education	202
33	Kiropoulos et al.(2004) [57]	Cross-sectional study		Greece, Anglo- Australians	Education, depression	292
34	Liddell et al.(2013) [58]	Cross-sectional study		Vietnam, Australian-born host country	Depression, education	12,164
35	Lies et al. (2019) [59]	Longitudinal study, follow-up period 2.5 years	From a large multicentre psychological service provider for refugees and asylum seekers	Participants were from 61 different countries	Depression	2703
36	Lin et al. (2016) [60]	Cross-sectional study		China, Australians	Loneliness, depression, education	119
37	Lumley et al.(2018) [61]	Cross-sectional study		Bhutan	Depression, education	148
38	Maldari et al. (2019) [62]	Cross-sectional study		Syria	Depression, smoking, obesity	455
39	Maneze et al. (2018) [63]	Cross-sectional study		Philippines	Hypertension, diabetes	58
40	May et al.(2014) [64]	Cross-sectional study		Iraqi and Sudanese participants	depression, education	97
41	Meng et al. (2014) [65]	Cross-sectional study		China	Hypertension, diabetes, depression	100
42	Menigoz et al. (2016) [66]	Longitudinal study, follow-up period 4 months	HILDA	Oceania (excluding Australia), North-West Europe, Southern & Eastern Europe, North Africa & The Middle East, South-East Asia, North-East Asia, Southern & Central Asia, Americas, Sub-Saharan Africa	Obesity, education	16,024
43	Momartin et al. (2004) [67]	Cross-sectional study		Bosnia	Depression	126
44	Nickerson et al. (2019) [68]	Longitudinal study, follow-up period 6 months	BNLA	Iraq, Afghanistan, Iran and Myanmar	Depression	1894
45	Oei et al. (2005) [69]	Cross-sectional study		Chile, El Salvador, Nicaragua, Argentina and Guatemala. Participants	Depression	101
46	Pasupuleti et al. (2016) [70]	Longitudinal study, follow-up period 6 years	HILDA Waves 3, 7 and 9	Australian-born, Asian born (India, the Philippines, Vietnam, China, Malaysia, Lebanon, Sri Lanka, Hong Kong, Indonesia and others	Diabetes, smoking, education, alcohol and alcohol use, physical inactivity	5485
47	Perusco et al. (2010) [71]	Cross-sectional study	A comprehensive social marketing campaign	Lebanon	Smoking, education	1102
48	Ponsford et al. (2020) [72]	Longitudinal study follow-up period 9 years	Traumatic brain injury rehabilitation programme at Epworth Healthcare	Participants were from 43 different countries	Traumatic brain injury, depression	206
49	Renzaho et al. (2014) [73]	Cross-sectional study		Sudan	Smoking, education, diabetes, obesity, alcohol use, hypertension	314
50	Renzaho et al. (2011) [74]	Cross-sectional		Ethiopia, Sudan, Eritrea, Somalia, Congo, Ghana, Rwanda and Nigeria	Diabetes, obesity, hypertension	49
51	Rowe et al. (2020) [75]	Cross-sectional		Southeast Asia, southern and eastern Europe, southern and central Asia, Northeast Asia, northern and western Europe (excluding the United Kingdom and Ireland), sub-Saharan Africa, and the Middle East, Oceania (excluding New Zealand), Australia, the Americas (excluding North America)	Smoking, alcohol use	22,696
52	Sahle et al. (2020) [76]	Longitudinal study, follow up period 11 years	HILDA	Australian-born, Asian born (India, the Philippines, Vietnam, China, Malaysia, Lebanon, Sri Lanka, Hong Kong, Indonesia. The remaining were born in other Asian countries that individually contributed less than 2.40% to the Asian-born sample	Alcohol use, physical inactivity, obesity	9916
53	Saltapidas and Ponsford, (2007) [77]	Cross-sectional study		China, Vietnam, Greece, and Italy, Hong Kong, Malta, and India, and one each from Czechoslovakia, Croatia, Germany, Lebanon, Singapore, Slovenia, Sri Lanka, Thailand, Ukraine, and Uruguay	Traumatic brain injury, education	70
54	Sanchez et al. (2020) [78]	Cross-sectional study		El Salvador, Colombia, and Chile	Smoking, diabetes, obesity, hypertension	382
55	Sarich et al. (2015) [75]	Longitudinal study, follow-up 3 years	45 and Up Study	Participants born in 14 different countries	Smoking, alcohol use, physical inactivity, obesity	26,4102
56	Schweitzer et al. (2011) [79]	Cross-sectional		India, Sri Lanka, Pakistan, Bangladesh, and Burma	Depression, education	70
57	Schweitzer et al. (2018) [80]	Cross-sectional		African countries (including Eritrea, Democratic Republic of Congo, Ethiopia, Sudan, South Sudan, Rwanda, Burundi, and Kenya), with other women coming from countries within South Asia (Afghanistan), West Asia (including Iran, Iraq, and Syria), and South-East Asia (including Myanmar and Thailand)	Depression, education	104
58	Shamshirgaran et al.(2019) [81]	Longitudinal study, follow-up 3 years	45 and Up Study	Southeast Asia, southern and eastern Europe, southern and central Asia, Northeast Asia, northern and western Europe (excluding the United Kingdom and Ireland), sub-Saharan Africa, and the Middle East, Oceania (excluding New Zealand), Australia, the Americas (excluding North America)	Hearing loss, diabetes	26,6848
59	Shamshirgaran et al. (2015) [82]	Longitudinal study, follow-up 3 years	45 and Up Study	Southeast Asia, southern and eastern Europe, southern and central Asia, Northeast Asia, northern and western Europe (excluding the United Kingdom and Ireland), sub-Saharan Africa, and the Middle East, Oceania (excluding New Zealand), Australia, the Americas (excluding North America)	Smoking, alcohol use, physical inactivity, diabetes	23,112
60	Shamshirgaran et al.(2013) [83]	Longitudinal study, follow-up 3 years		Australia, New Zealand, Rest of Oceania and Antarctica, United Kingdom, Germany, Netherlands, Italy, Greece, Rest of Europe, Egypt, Lebanon, Rest of the Middle East and North Africa, Vietnam, Philippines, China, India, Sri Lanka, Rest of Asia, Americas, and South Africa	Obesity, smoking, physical inactivity, diabetes	266,848
61	Silove et al. (2010) [84]	Cross-sectional		Bosnia	Depression	126
62	Silove et al.(2007) [85]	Longitudinal study, follow-up 11.3 months	A prospective cohort study of asylum seekers	Iran, Ghana, Iraq, Zimbabwe, Afghanistan and China	Depression, education	62
63	Slewa-Younan et al., (2020) [86]	Cross-sectional		Iraq, Lebanon	Education	52
64	Slewa-Younan et al.(2017) [87]	Cross-sectional		Afghan	Depression, education	150
65	Slewa-Younan et al. (2014) [88]	cross-sectional		Iraq	Depression, education	79
66	Stanaway et al. (2020) [89]	Longitudinal study, follow-up period 8 years	Concord Health and Ageing in Men Project (CHAMP)	Italian-born and Australian-born	Obesity, smoking, physical inactivity, depression, education, hypertension	1183
67	Stanaway et al. (2011) [90]	Longitudinal study, follow-up period 2.5 years	CHAMP	Italian-born and Australian-born	Education, depression	1183
69	Steel et al.(2005) [91]	Cross-sectional		Vietnam	Depression, education, alcohol use	9572
70	Straiton et al. (2014) [92]	Longitudinal study, follow-up period 3 years	North-West Adelaide Health Study (NWAHS)	over 40 different countries	Depression, education	4056
71	Tan et al.(2013) [93]	Longitudinal study, follow-up period 3 years	A multi-racial Australian community: the Fremantle Diabetes Study	Anglo-Celt and Asians	Diabetes, obesity, hypertension, education, smoking	840
72	Tang et al.(2009) [94]	Cross-sectional study		China	Depression, education	161
73	Taylor et al. (2018) [21]	Cross-sectional study	An Italian (PASSI), and an Australian (SAMSS) Risk Factor Surveillance System	Italy	Hypertension, diabetes, smoking education, alcohol, and obesity	206,726
74	Tran et al. (2015) [95]	Longitudinal study, follow-up period 3 years	45 and Up Study	Vietnam	Smoking, education, diabetes, obesity, alcohol use	797
75	Tran et al. (2014) [96]	Longitudinal study, follow-up period 3 years	45 and Up Study	Vietnam	Obesity, smoking, education, physical inactivity, diabetes	197,653
76	Vromans et al. (2020) [97]	Longitudinal study, follow-up period 20 months		African nations included Eritrea, Democratic Republic of Congo, Ethiopia, Sudan, South Sudan, Rwanda, Burundi, Somalia, and Kenya Afghanistan, Iran, Iraq, and Myanmar	Depression	83
77	Weber et al. (2011) [98]	Longitudinal study, follow-up period 3 years		Participants born in 162 different countries	Smoking, education	101,984
78	Wu et al. (2021) [99]	Longitudinal study, follow-up period 3.5 years	BNLA	Participants born in 35 different countries	Loneliness, education	1929
79	Wyk et al. (2012) [100]	Longitudinal study, follow-up period 3.09 months	A longitudinal study of mental health in refugees from Burma: The impact of therapeutic interventions	Burma	Depression, education	62

Building a New Life in Australia Survey (BNLA); Cognitive Behaviour Therapy (CBT); Concord Health and Ageing in Men Project (CHAMP); Household, Income and Labour Dynamics in Australia (HILDA) The Health In Men Study (HIMS); Humanitarian Migrants (HMs); Longitudinal Survey of Immigrants to Australia (LSIA); Melbourne Collaborative Cohort Study (MCCS); North West Adelaide Health Study (NWAHS); Randomised controlled trial (RCT); Victorian Population Health Survey (VPHS)

**Table 2:** Number of studies reporting higher/lower prevalence of modifiable risk factors in migrants

Lancet Commission’s 12 risk factors	Number (K)	Number higher (k)	Number lower (k)	Number NR (k)
Depression	32	3	0	29
Smoking	26	3	3	20
Less education	43	9	3	31
Hearing loss	1	0	1	0
Traumatic brain injury	2	0	0	2
Hypertension	14	2	3	9
Alcohol consumption	14	1	7	6
Obesity/overweight	20	2	5	13
Social isolation	6	1	0	5
Physical inactivity	21	8	0	13
Diabetes Mellitus	19	8	0	11
Air pollution	0	0	0	0

*K*  Number of studies; *Higher/Lower rate* Number of studies reporting migrants and refugees have a higher/lower prevalence of the risk factor than the host population *NR* not reported

### Depression

Thirty-two articles reported on depression. Of these, 20 (62.5%) were cross-sectional in design, 10 (31.2%) were longitudinal and two (6.2%) were randomized controlled trials (RCTs). Sample sizes varied from small (n = 55) in a study of Turkish, Iranian, and Persian women in metropolitan Sydney [42] to a very large sample (n = 115,988) drawn from the 45 and Up Study [54]. Depression prevalence ranged from 7% among 12,164 Vietnamese migrants from the Mekong Delta [58] to 76% among Chinese residents living in ethno-specific nursing homes (NHs) and mainstream NHs in Sydney [43]. Results from a cross-sectional analysis from migrants in Chile, El Salvador, Nicaragua, Argentina and Guatemala indicated the relationship between depression and cognition was bidirectional [69]. Choi et al. [34] found that there was a significant reduction in symptoms of depression among controls compared with treatment group participants (n = 55) [34].

Three studies revealed that migrants had higher prevalence of depression than non-immigrant Australians [57, 65, 67, 79, 84, 85, 87–90, 92, 97, 100–102]. Longitudinal studies had timeframes ranging from 11.3 months [85] to 8 years [87]. Overall, there appears to be a higher prevalence of depression in refugee and migrant and asylum seeker populations than observed in non-immigrant Australians (Fig. 2). Longitudinal studies show some exacerbation of depressive symptoms through the processes involved in gaining asylum [85].

### Smoking

Twenty-six articles reported on smoking. From these studies, nine (24.6%) were cross-sectional and seventeen (65.4%) were longitudinal. Sample sizes varied from small (n = 55) [42] to very large (n = 266,848) [81]. Smoking prevalence ranged from 6.8% among 382 Latin American Spanish-speaking patients in Brisbane [78] to 45% among 382 male Chinese migrants in metropolitan Sydney [51]. Three studies indicated that the prevalence of smoking was higher among non-immigrant Australian participants than among foreign-born participants [20, 50, 70]. Another three studies found smoking prevalence was higher among migrants than non-immigrant Australians [47, 52, 102]. The disparities across studies in terms of smoking rates are illustrated in Fig. 2. Smoking in the foreign-born populations was associated with older age, male gender, lower work status, ethnicity and country of origin and length of stay in Australia, poor health, mental health disorders, and an increased risk of deaths [26, 36, 42, 49, 50, 53, 54].

### Education

Forty-three articles reported on education. Of these, twenty-one (48.8%) were cross-sectional, twenty (46.5%) were longitudinal, and two (4.6%) were RCTs. The sample sizes varied from small (n = 52) in a study of Arabic speaking religious and community leaders based in Southwestern Sydney [86] to very large (n = 266,848) [87]. Follow-up periods for the longitudinal studies ranged from 3 years among Vietnam-born Australians [95] to 11 years among 12,634 individuals from The Household, Income and Labour Dynamics in Australia (HILDA) [53]. Figure 2 illustrates that fifteen studies showed the majority of migrant and refugee samples had limited primary school/some secondary school education [20, 29, 32, 42, 43, 45, 49, 55, 56, 61, 80, 95, 99, 103, 104]. Nine studies indicated migrant populations had lower educational attainment compared to non-immigrant Australians [20, 29, 34, 38, 39, 45, 47, 53, 57, 58, 64, 86, 91, 95, 96, 102]. Two studies demonstrated that, compared to non-immigrant Australians, the percentage of university educated participants was significantly higher among the Asian participants [50, 53]. Further, Tang et al. [94] found the percentage of participants with tertiary or equivalent education was significantly higher among Chinese-born migrants compared with non-immigrant Australians [94]. However, there were no significant differences in education levels between Vietnamese-born and non-immigrant Australians [96]. Ponsford et al. [72] found no significant differences between CALD and non-CALD participants in education levels [72]. Chou et al. (2007) found that there was an association between education levels and the General Health Questionnaire scores, depression, gender, the country of birth and ethnicity [77].

### Hearing Loss

One study reported on hearing loss. In this study, foreign-born participants had a lower rate of hearing loss than Australian participants [81]. Among participants with diabetes, this longitudinal study found hearing loss was significantly associated with age, male gender and those with lower income and low education levels [81].

### Traumatic Brain Injury

Two articles reported on TBI. Sample sizes range from 70 [61] to 206 [56]. Overall, there is a lack of consensus on the incidence or prevalence of TBI in Australian migrants and refugees. However, research shows that CALD groups have poorer functional outcomes following TBI than those from a non-CALD background [72, 74].

### Hypertension

Fourteen articles reported on hypertension. Of these, five (45.4%) were cross-sectional and nine (54.6%) were longitudinal. The sample sizes varied from small (n = 49) [74] to very large (n = 263,356) [44]. Hypertension prevalence ranged from 12.4% [73] to 91% [63]. Follow-up period ranged from 2 years [44] to 5.9 years [54]. Two studies found migrant and refugee populations had a higher prevalence of hypertension compared to non-immigrant Australians [20, 44], while three studies found the prevalence was higher in non-immigrant Australians [39, 52, 93] (Fig. 2). Hypertension prevalence was associated with older age, longer duration of Australian residence and being born in Pacific Islands, Southeast Asia and Italy [41, 52, 73, 103].

### Alcohol Consumption

Fourteen articles reported on alcohol. Of these, four (28.6%) were cross-sectional, and 10 (71.4%) longitudinal. Samples varied from medium-sized (n = 225) [29] to very large (n = 266,848) [81]. The prevalence of alcohol use ranged from 6.1% among Vietnamese-born [29] to 89.0% among 22,696 non-Indigenous Australians [105]. Seven studies found foreign-born participants had a lower prevalence of alcohol consumption compared to non-immigrant Australians [20, 40, 70, 75, 82, 91, 105] (Fig. 2). Foreign-born Asians such as Chinese, Vietnamese, Philippines, Lebanese had the lowest rates of alcohol consumption [40].

### Obesity

Twenty articles reported on obesity. Of these, seven (35%) were cross-sectional, and thirteen (65%) were longitudinal. Sample sizes varied from small (n = 49) [74] to very large (n = 263,356) [44]. In longitudinal studies, the follow-up periods ranged from two years [44] to 10 years [76]. The prevalence of obesity ranged from 20.8% [20] to 84.0% [42].

Six studies found the prevalence of obesity was higher among non-immigrant Australians than migrant, refugee and asylum seeker populations [44, 46, 52, 75, 96] (Fig. 2). Two studies found higher prevalence in migrant populations [34, 72]. The prevalence of obesity was significantly lower among first generation East Asian, South Asian and North-West European migrants than non-immigrant Australians. However, South Europeans had significantly higher body weights than non-immigrant Australians [46].

### Social Isolation

Six articles reported on social isolation. Of these, two (33.3%) were cross-sectional, and four (66.6%) longitudinal. Sample sizes varied from small (n = 66) [45] to very large (n = 263,356) [44]. Only one study reported on differences between migrants and non-immigrant Australian population, where the prevalence of loneliness was significantly higher among 59 Chinese migrants (49%) than 60 non-immigrant Australian participants (13%) [60].

### Physical Inactivity

Of the 21 articles reporting physical inactivity, six (28.6%) were cross-sectional and fifteen (71.4%) were longitudinal. Sample sizes varied from small (n = 66) [78] to very large (n = 266,848) [81]. The prevalence of physical inactivity ranged from 21.7% among Asians in the HILDA study [70] to 72.3% among resettled Afghan refugees residing in Launceston [45]. In longitudinal studies, the follow-up periods ranged from six months [33] to 11 years [46]. Eight studies found that migrants and refugees living in Australia were more likely to be physically inactive compared to non-immigrant Australians [37, 39, 44, 52, 70, 82, 96, 102]. Figure 2 illustrates none of the included studies found physical inactivity was higher in non-immigrant Australians.

### Diabetes

Nineteen articles reported on diabetes, of which eight (42.1%) were cross-sectional, and eleven (57.9%) longitudinal, with follow-up ranging from three [93] to eight years [34]. Sample sizes varied from small (n = 49) [80] to very large (n = 266,848) [81]. Diabetes prevalence ranged from 1.5% [93] to 33% [99]. Figure 2 illustrates that eight of the included studies found migrants and refugees had a higher prevalence of diabetes than their non-immigrant Australian counterparts [20, 28, 34, 48, 52, 70, 95, 96]. The prevalence varied from 1.5% to 15.5% among non-immigrant Australians participants and from 1.5 to 33.0% among migrant, refugee, asylum seeker groups [20, 28, 34, 44, 52, 63, 70, 93, 106].

## Discussion

This systematic review sought to explore the reported prevalence of modifiable dementia risk factors [18] among migrant, refugee and asylum seeker populations residing in Australia. We reviewed published literature that reported rates of depression, smoking, educational attainment, hearing loss, TBI, hypertension, excessive alcohol consumption, obesity, social isolation, physical inactivity and diabetes among the target population. We found considerable variation in the degree to which prevalence of lifestyle and behaviour-related dementia risk factors in the target samples has been reported. Risk factor prevalence was associated with participants’ demographic characteristics, culture of origin, life course events, country of birth, age at arrival and length of stay in Australia.

The prevalence of depression was higher among migrant, refugee and asylum seeker populations than non-immigrant Australians [90, 97, 102]. Our findings are similar to other studies in 10 countries in North America (Canada and the US), Latin America (Brazil, Chile, and Mexico), Europe (Czech Republic, Germany, the Netherlands, and Turkey), and Asia (Japan) [107, 108]. Several migration-related factors may have contributed to this higher prevalence. Migrant populations may have experienced prolonged exposure to war and pre- and post-resettlement stressors [75]. Further, there is an association between depression and post-migration stressors in the new environment. As a result, migrants are at a heightened risk of experiencing depression [109, 110]. Encouragingly it appears that people with higher levels of depression are more likely to be in treatment, and that both information-based and therapeutic (e.g. CBT) interventions targeting depression have been shown to effectively reduce symptoms among Australian refugees and migrants [111].

There were marked disparities across studies investigating the prevalence of smoking among migrants and refugees compared with non-immigrant Australian populations [20, 39, 50, 52, 102, 112–115]. A lower prevalence of smoking among migrants may to some extent be explained by the characteristics of participants, religious beliefs, social, economic and cultural factors that influence arrived migrants from non-English speaking countries taking up smoking [76]. On the other hand, a higher prevalence of smoking could be attributed to increased duration of residence or an accumulation of stress-inducing events since resettlement [116]. Moreover, discrimination was associated with unhealthy behaviours such as smoking and substance use among refugees, migrants and asylum seekers [117]. We found smoking was associated with older age, male gender, higher body mass index, longer duration of residence in Australia, poorer health, mental health disorders, racial ethnic origin and an increased risk of death [26, 36, 42, 49, 50, 53, 54].

Educational attainment was lower among foreign-born population than Australian participants in nine studies [20, 38, 39, 47, 58, 64, 71, 91, 96, 102], but higher among migrants in three [50, 53, 94]. Overall, compared with non-immigrant Australians, there was higher educational attainment among migrants from India and China. However, migrants with a low level of tertiary education were more likely to enter higher education and get a degree than their non-immigrant Australian counterparts [118]. Education empowers by giving foreign-born populations the knowledge and skills to attain a good job and a better life [119], and is thus a viable target for dementia risk reduction interventions.

In one study of participants with diabetes, hearing loss prevalence was lower in foreign-born participants than non-immigrant Australians [81]. This is supported by a US study which revealed a lower rate of hearing impairment among non-Hispanic Black or Mexican Americans than Non-Hispanic white participants [120]. It is likely that many migrants with diabetes have more difficulty than non-immigrant Australians in seeking and receiving effective healthcare due to language difficulties, cultural differences, health system barriers and health literacy deficits [121].

Two studies reported on TBI among CALD and non-CALD participants. Compared with non-CALD participants, CALD participants reported poorer functional outcomes following TBI, including lower physical independence, cognitive independence, mobility, and participation in occupational and social activities [72]. These results were in line with prior work conducted by Saltapidas and Ponsford [77] who suggested the difference may be due to unobserved cultural differences beyond the prevalence of TBI incidence [77] other than differences in TBI prevalence between CALD and non-CALD groups [72].

There were marked differences in the prevalence of hypertension among migrant populations compared with host populations [20, 39, 44, 52, 93]. Differences in ethnicity might explain lower rates of hypertension [122]. For example, hypertension risk is lower in Asian population than the host population [93]. Guo et al. [44] demonstrated a longer duration of residence in Australia was associated with an increased risk for hypertension among most Asian migrant participants [44], which is consistent with results from a study of Chinese migrants in Canada [123]. The higher prevalence of hypertension among foreign-born than non-immigrant Australians could be due to the increase in the prevalence of hypertension in low- and middle-income countries [124]. There was a significant lowering of blood pressure, an increased knowledge of hypertension and greater compliance with medical treatment six weeks after the intervention [125]. Therefore, the high rates of hypertension among foreign-born populations from those countries may merely reflect the rising rates in the participants’ countries of origin.

Substantial disparities were noted in the reported prevalence of alcohol consumption in foreign-born populations compared with non-immigrant Australians as well some differences by racial group [20, 70, 75, 82, 91, 105]. These results are similar to those from the US [126]. Notably, foreign-born Italian, Irish, New Zealand, Scandinavian, and British populations had alcohol consumption rates higher than non-immigrant Australians suggesting they may be at a higher risk of alcohol abuse. Foreign-born Asians such as Chinese, Vietnamese, Philippines, Lebanese had the lowest rates of alcohol abuse [20, 40, 82]. Alcohol abuse in the foreign-born populations was associated with male gender, unemployment, ethnicity, country of origin and length of stay in Australia [40]. The considerable variability across ethnicity and country of origin in alcohol use likely presents similar patterns in a multicultural society like Australia.

We found the majority of studies reported higher prevalence of obesity in non-immigrant Australian participants than foreign-born participants [44, 52, 75, 93, 96]. However, two studies found the prevalence was higher in migrant populations [96, 102]. This is consistent with previous findings in the US [127]. A number of contributing factors have been postulated to explain these variations, such as behavioural, genetic, cultural, contextual, and systemic factors [128]. Foreign-born East Asian, South Asian and North-West European migrants had lower prevalence of obesity than non-immigrant Australian individuals [46], while male migrants born in North Africa/Middle East, Oceania and Southern and Eastern European countries had higher prevalence of obesity compared with their non-immigrant Australian counterparts [83]. Obesity trends among foreign-born participants were associated with length of residence in Australia, female gender, diabetes, mental health illness and older age [46, 73, 76, 83, 93, 129]. This might also be related to acculturation such as lifestyle factors, such as diet and exercise, under the influence of Australian culture [118].

Our study revealed foreign-born participants experience higher levels of loneliness than non-immigrant Australians [60]. These results are comparable to those reported in a study conducted in Canada [27]. Poor general health, post-traumatic stress disorder, severe mental illness, length of residence, depressive symptoms, psychological distress and economic stressors are established correlates of loneliness and isolation among foreign-born individuals [32, 33, 45, 99]. Previous research has shown the prevalence of loneliness in foreign-born individuals increased with a longer duration of residence in Australia [49]. These findings provide further evidence for the need to improve social interactions with friends and family members in preventing or improving loneliness and isolation among migrant, refugee and asylum seeker populations.

Physical inactivity rates were higher among foreign-born populations than non-immigrant Australians [39, 44, 52, 70, 96, 102]. Physical inactivity was associated with a longer duration of residence in Australia, depression, diabetes, post-war trauma, economic stressors, female gender, diabetes, and risk of mortality. Foreign-born Southeast Asia, Other Asia, Oceania, the Middle East, and Southern & Eastern Europe had higher prevalence of physical inactivity than non-immigrant Australians [26, 27, 29–31, 37, 44, 45, 82, 106, 129, 130]. People in marginalised groups often face common social, economic and individual problems which make having access to leisure time physical activity more difficult, leading to lower participation in leisure activities and physical activities. Our findings have implications for improving the health and well-being of foreign-born populations. They could be encouraged to adopt a healthy lifestyle in Australia including increased participation in leisure activities, healthy eating, and meaningful physical activities [131].

In all studies we identified, the prevalence of diabetes was higher among foreign-born populations than non-immigrant Australians [20, 28, 48, 52, 70, 95, 96, 102]. These results are consistent with a previous study conducted in the US suggesting that migrants from India and Central America have a higher prevalence of diabetes than White Americans [132]. Physical inactivity is an important determinant of chronic diseases such as diabetes, cardiovascular diseases, or obesity among these populations and the higher prevalence of physical inactivity among migrants in this study may have contributed to a higher prevalence of diabetes [39, 52, 70, 87]. Studies from foreign-born populations in Australia suggest that diabetes is associated with poorer general health, mental health disorder, length of residence, age, obesity, smoking, physical inactivity, an increased risk of developing kidney disease and death [26, 44, 48, 54, 75, 83, 93, 102, 103].

This is the first review to systematically investigate the evidence regarding modifiable dementia risk factors among migrants, refugees and asylum seekers. We present a double-screened, quality-checked synthesis of peer-reviewed literature regarding risk factor prevalence in migrant populations, in the hopes of supporting future research targeting risk factors that are higher in-migrant populations. This systematic review has several limitations. Firstly, searches were restricted to those available electronically and in English. Secondly, there was wide variability in the methods used in the included studies for data collection and reporting. Third, this review identified some possible life and environmental factors that are highly related to migrants, such as discrimination, cultural barriers, health literacy deficits and the period of residence in the host country. These factors may be associated with higher dementia risk and may impact research participation, but could not be captured in this systematic review. Furthermore, differences in designs, participants, and settings and outcomes were identified. Substantial methodological heterogeneity was identified in data extraction, rendering meta-analysis unapplicable with our current protocol. Lastly, none of the studies explicitly identified and examined outcome variables framed as modifiable dementia risk factors. Instead, rates for the variables of interest were extracted and reported per study.

Our recommendation for future research is targeted recruitment among migrant, refugee and asylum seeker populations and explicit assessment of the presence and extent of modifiable dementia risk factors. These risk factors are clear candidates for targeted public health interventions and include depression, hypertension, social isolation, physical inactivity, diabetes, obesity, and smoking. However, more research is needed for cognitive activity, biomedical factors (such as prolonged stress, diet, inadequate sleep and various health conditions) and environmental risk. Importantly this research should be conducted with a sample sufficiently large and diverse for examining the moderating role of country of origin, refugee vs migrant status, and time resettled in Australia. This information can then be used to guide and inform public health initiatives aiming to target and reduce identified risk factors impacting these vulnerable segments of Australia’s population.

## Conclusions

Compared with non-immigrant Australians- people, this systematic review found a higher prevalence among migrants, refugees and asylum seekers in the dementia risk factors of depression, social isolation, physical inactivity and diabetes. The modifiable dementia risk factors were reported to be related to participants’ sociodemographic factors, physical and mental health, cultures, experience, or accumulation of effects over the life course the country of birth, age at arrival in the host country and the length of stay in the host country. Due to substantial heterogeneity between studies’ reporting, methods and primary outcomes, it was difficult to synthesize the findings across each risk factor. However, our review illustrates the need for a concerted effort to better understand patterns of dementia risk lifestyle and behavioral factors to inform the design and implementation of risk reduction strategies for migrants, refugees and asylum seekers who live in Australia.

## Supplementary Information

Below is the link to the electronic supplementary material.

Supplementary file1 (DOCX 84 kb)

Supplementary file2 (XLSX 23 kb)
