# Supplementary material for: Modifiable Risk Factors for Dementia Among Migrants, Refugees and Asylum Seekers in Australia: A Systematic Review
Source: J Immigr Minor Health. 2023 Jan 18;25(3):692–711. doi: 10.1007/s10903-022-01445-2 (PMC10212820; doi:10.1007/s10903-022-01445-2)
Supplement: Supplementary file 1 — Supplementary file1 (DOCX 84 kb) [file 10903_2022_1445_MOESM1_ESM.docx]

**Supplementary materials for:**

**Table of contents.**

**1.** **Table S1: PRISMA Checklist ..................................................................................................................................................................P.2-4**

**1. Table S2: Search terms .............................................................................................................................................................................P.5-6**

**3. Table S3: NIH Quality Assessment** **of Controlled Intervention Studies...............................................................................................P.7-8**

**4. Table S4: NIH Quality Assessment** **Tool for Observational Cohort and Cross-sectional Studies.....................................................P.9-22**

**Supplementary Table S1:** PRISMA Checklist for Systematic Reviews and Meta Analyses. From: Moher et al. (2009)

| # | Section/topic | Checklist item | Reported on page # |
| --- | --- | --- | --- |
|  | **TITLE** |  |  |
| 1 | Title | Identify the report as a systematic review, meta-analysis, or both. | 1 |
|  | **ABSTRACT** |  |  |
| 2 | Structured summary | Provide a structured summary including, as applicable: background; objectives; data sources; study eligibility criteria, participants, and interventions; study appraisal and synthesis methods; results; limitations; conclusions and implications of key findings; systematic review registration number. | 2 |
|  | **INTRODUCTION** |  |  |
| 3 | Rationale | Describe the rationale for the review in the context of what is already known. | 3-4 |
| 4 | Objectives | Provide an explicit statement of questions being addressed with reference to PICOS. | 4 |
|  | **METHODS** |  |  |
| 5 | Protocol and registration | Indicate if a review protocol exists, if and where it can be accessed (e.g., Web address), and, if available, provide registration information including registration number. | 5-6 |
| 6 | Eligibility criteria | Specify study characteristics (e.g., PICOS, length of follow-up) and report characteristics (e.g., years considered, language, publication status) used as criteria for eligibility, giving rationale. | 5 |
| 7 | Information sources | Describe all information sources (e.g., databases with dates of coverage, contact with study authors to identify additional studies) in the search and date last searched. | 4 |
| 8 | Search | Present full electronic search strategy for at least one database, including any limits used, such that it could be repeated. | 5 |
| 9 | Study selection | State the process for selecting studies (i.e., screening, eligibility, included in systematic review, and, if applicable, included in the meta-analysis). | 5 |
| 10 | Data collection process | Describe method of data extraction from reports (e.g., piloted forms, independently, in duplicate) and any processes for obtaining and confirming data from investigators. | 5 |
| 11 | Data items | List and define all variables for which data were sought (e.g., PICOS, funding sources) and any assumptions and simplifications made. | 5 |
| 12 | Risk of bias in individual studies | Describe methods used for assessing risk of bias of individual studies (including specification of whether this was done at the study or outcome level), and how this information is to be used in any data synthesis. | 5 |
| 13 | Summary measures | State the principal summary measures (e.g., risk ratio, difference in means). | 6 |
| 14 | Synthesis of results | Describe the methods of handling data and combining results of studies, if done, including measures of consistency (e.g., I2) for each meta-analysis. | 6 |
| 15 | Risk of bias across studies | Specify any assessment of risk of bias that may affect the cumulative evidence (e.g., publication bias, selective reporting within studies). | 6 |
| 16 | Additional analyses | Describe methods of additional analyses (e.g., sensitivity or subgroup analyses, meta-regression), if done, indicating which were pre-specified. | 6 |
|  | **RESULTS** |  |  |
| 17 | Study selection | Give numbers of studies screened, assessed for eligibility, and included in the review, with reasons for exclusions at each stage, ideally with a flow diagram. | 7 |
| 18 | Study characteristics | For each study, present characteristics for which data were extracted (e.g., study size, PICOS, follow-up period) and provide the citations. | 7- 11 |
| 19 | Risk of bias within studies | Present data on risk of bias of each study and, if available, any outcome level assessment (see item 12). | 7- 11 |
| 20 | Results of individual studies | For all outcomes considered (benefits or harms), present, for each study: (a) simple summary data for each intervention group (b) effect estimates and confidence intervals, ideally with a forest plot. | 7- 11 |
| 21 | Synthesis of results | Present results of each meta-analysis done, including confidence intervals and measures of consistency. | 7- 11 |
| 22 | Risk of bias across studies | Present results of any assessment of risk of bias across studies (see Item 15). | 7- 11 |
| 23 | Additional analysis | Give results of additional analyses, if done (e.g., sensitivity or subgroup analyses, meta-regression [see Item 16]). | 7- 11 |
|  | **DISCUSSION** |  |  |
| 24 | Summary of evidence | Summarize the main findings including the strength of evidence for each main outcome; consider their relevance to key groups (e.g., healthcare providers, users, and policy makers). | 13-17 |
| 25 | Limitations | Discuss limitations at study and outcome level (e.g., risk of bias), and at review-level (e.g., incomplete retrieval of identified research, reporting bias). | 18-19 |
| 26 | Conclusions | Provide a general interpretation of the results in the context of other evidence, and implications for future research. | 19 |
|  | **FUNDING** |  |  |
| 27 | Funding | Describe sources of funding for the systematic review and other support (e.g., supply of data); role of funders for the systematic review. | 19 |

**Key:** PICOS = Participants, Interventions, Comparisons, Outcomes, and Study design

**Supplementary Table S2:** **Search terms**

| **n** | **Search terms** | **MEDLINE [UPDATED on 2 March] Found articles (n)** | **CINAHL [UPDATED on 2 March]** | **PUBMED [UPDATED on 2 March]** |
| --- | --- | --- | --- | --- |
| 1 | refugee* OR migrant* OR immigrant* OR asylum seeker* OR CALD OR linguistically diverse | 64,649 | 35,054 | 67,875 |
| 2 | depression | 441,599 | 177,359 | 531,540 |
| 3 | smoking OR tobacco | 364,049 | 123,883 | 382,025 |
| 4 | education level | 48,728 | 23,267 | 218,631 |
| 5 | hearing loss OR hearing impairment | 97,075 | 35,785 | 98,246 |
| 6 | traumatic brain injury OR head injury OR brain injury | 134,379 | 44,822 | 272,450 |
| 7 | hypertension OR blood pressure | 831,504 | 176,813 | 991,222 |
| 8 | alcohol* | 469,564 | 109,853 | 457,396 |
| 9 | obesity OR overweight OR obese | 395,529 | 143,789 | 398,103 |
| 10 | social isolation OR loneliness | 27,326 | 17,040 | 37,684 |
| 11 | physical inactivity OR sedentary | 43,226 | 19,401 | 45,109 |
| 12 | air pollution | 63,601 | 10,902 | 83,254 |
| 13 | diabetes | 709,131 | 222,687 | 801,858 |
| 14 | AB Australia* | 124,990 | 63,192 | 147,747 |
| 15 | 2 OR 3 OR 4 OR 5 OR 6 OR 7 OR 8 OR 9 OR 10 OR 11 OR 12 OR 13 | 3,068,532 | 921,065 | 3,638,845 |
| 16 | 1 AND 15 | 10,519 | 6,303 | 13,350 |
| 17 | 14 AND 16 | 520 | 301 | 663 |

**Supplementary Table S3:** NIH Study Quality Assessment Tool for studies included in the systematic review and meta-analysis: Controlled Intervention Studies

| Quality Assessment of Controlled Intervention Studies | | Choi et al. (2012) | Kiropoulos et al. (2012) |
| --- | --- | --- | --- |
| 1 | Was the study described as randomized, a randomized trial, a randomized clinical trial, or an RCT? | Y | Y |
| 2 | Was the method of randomization adequate (i.e., use of randomly generated assignment)? | Y | Y |
| 3 | Was the treatment allocation concealed (so that assignments could not be predicted)? | Y | Y |
| 4 | Were study participants and providers blinded to treatment group assignment? | N | N |
| 5 | Were the people assessing the outcomes blinded to the participants' group assignments? | N | N |
| 6 | Were the groups similar at baseline on important characteristics that could affect outcomes (e.g., demographics, risk factors, co-morbid conditions)? | Y | Y |
| 7 | Was the overall drop-out rate from the study at endpoint 20% or lower of the number allocated to treatment? | N | N |
| 8 | Was the differential drop-out rate (between treatment groups) at endpoint 15 percentage points or lower? | Y | Y |
| 9 | Was there high adherence to the intervention protocols for each treatment group? | Y | Y |
| 10 | Were other interventions avoided or similar in the groups (e.g., similar background treatments)? | NR | NR |
| 11 | Were outcomes assessed using valid and reliable measures, implemented consistently across all study participants? | Y | Y |
| 12 | Did the authors report that the sample size was sufficiently large to be able to detect a difference in the main outcome between groups with at least 80% power? | Y | Y |
| 13 | Were outcomes reported or subgroups analyzed prespecified (i.e., identified before analyses were conducted)? | NA | NA |
| 14 | Were all randomized participants analyzed in the group to which they were originally assigned, i.e., did they use an intention-to-treat analysis? | Y | Y |

**Key:** Y = Yes; N = No; CD = cannot determine; NA = not applicable; NR = not reported

**Supplementary Table S4:** NIH Study Quality Assessment Tool for studies included in the systematic review and meta-analysis: Observational Cohort and Cross-Sectional Studies

| Quality Assessment Tool for Observational Cohort and Cross-Sectional Studies | | Almeida et al. (2010) | Astell-Burtet al. (2013) |
| --- | --- | --- | --- |
| 1 | Was the research question or objective in this paper clearly stated? | Y | Y |
| 2 | Was the study population clearly specified and defined? | Y | Y |
| 3 | Was the participation rate of eligible persons at least 50%? | N | N |
| 4 | Were all the subjects selected or recruited from the same or similar populations (including the same time period)? Were inclusion and exclusion criteria for being in the study prespecified and applied uniformly to all participants? | Y | N |
| 5 | Was a sample size justification, power description, or variance and effect estimates provided? | Y | Y |
| 6 | For the analyses in this paper, were the exposure(s) of interest measured prior to the outcome(s) being measured? | Y | Y |
| 7 | Was the timeframe sufficient so that one could reasonably expect to see an association between exposure and outcome if it existed? | N | N |
| 8 | For exposures that can vary in amount or level, did the study examine different levels of the exposure as related to the outcome (e.g., categories of exposure, or exposure measured as continuous variable)? | Y | Y |
| 9 | Were the exposure measures (independent variables) clearly defined, valid, reliable, and implemented consistently across all study participants? | N | N |
| 10 | Was the exposure(s) assessed more than once over time? | Y | Y |
| 11 | Were the outcome measures (dependent variables) clearly defined, valid, reliable, and implemented consistently across all study participants? | N | N |
| 12 | Were the outcome assessors blinded to the exposure status of participants? | Y | Y |
| 13 | Was loss to follow-up after baseline 20% or less? | N | N |
| 14 | Were key potential confounding variables measured and adjusted statistically for their impact on the relationship between exposure(s) and outcome(s)? | N | N |

| Quality Assessment Tool for Observational Cohort and Cross-Sectional Studies | | Chen et al. (2017) | Chou et al. (2007) | Christopoulou et al. (2014) | Dassanayake et al. (2001) | Drummond et al. (2010) | El Masri et al. (2019) | Feng al. (2014) |
| --- | --- | --- | --- | --- | --- | --- | --- | --- |
| 1 | Was the research question or objective in this paper clearly stated? | Y | Y | Y | Y | Y | Y | Y |
| 2 | Was the study population clearly specified and defined? | Y | Y | N | N | N | Y | N |
| 3 | Was the participation rate of eligible persons at least 50%? | Y | Y | NR | NR | Y | N | N |
| 4 | Were all the subjects selected or recruited from the same or similar populations (including the same time period)? Were inclusion and exclusion criteria for being in the study prespecified and applied uniformly to all participants? | Y | Y | Y | Y | Y | Y | Y |
| 5 | Was a sample size justification, power description, or variance and effect estimates provided? | N | Y | NR | N | Y | N | Y |
| 6 | For the analyses in this paper, were the exposure(s) of interest measured prior to the outcome(s) being measured? | N | N | N | NA | NA | NR | NR |
| 7 | Was the timeframe sufficient so that one could reasonably expect to see an association between exposure and outcome if it existed? | Y | Y | Y | NA | NA | Y | Y |
| 8 | For exposures that can vary in amount or level, did the study examine different levels of the exposure as related to the outcome (e.g., categories of exposure, or exposure measured as continuous variable)? | Y | Y | N | N | N | N | N |
| 9 | Were the exposure measures (independent variables) clearly defined, valid, reliable, and implemented consistently across all study participants? | Y | Y | Y | Y | Y | Y | Y |
| 10 | Was the exposure(s) assessed more than once over time? | N | N | N | NA | NA | N | N |
| 11 | Were the outcome measures (dependent variables) clearly defined, valid, reliable, and implemented consistently across all study participants? | Y | Y | Y | Y | Y | Y | Y |
| 12 | Were the outcome assessors blinded to the exposure status of participants? | N | N | N | N | N | N | N |
| 13 | Was loss to follow-up after baseline 20% or less? | N | N | NR | NA | NA | N | N |
| 14 | Were key potential confounding variables measured and adjusted statistically for their impact on the relationship between exposure(s) and outcome(s)? | Y | Y | N | Y | N | Y | Y |

| Quality Assessment Tool for Observational Cohort and Cross-Sectional Studies | | Gallegos et al. (2019) | Gholizadeh al. (2009) | Goh et al. (2010) | Guo et al. (2005) | Hamrah et al. (2020) | Hauck et al. (2010) | Hodge al. (2004) |
| --- | --- | --- | --- | --- | --- | --- | --- | --- |
| 1 | Was the research question or objective in this paper clearly stated? | Y | Y | Y | Y | Y | Y | Y |
| 2 | Was the study population clearly specified and defined? | Y | N | Y | Y | Y | Y | N |
| 3 | Was the participation rate of eligible persons at least 50%? | NR | NR | NR | N | Y | NR | Y |
| 4 | Were all the subjects selected or recruited from the same or similar populations (including the same time period)? Were inclusion and exclusion criteria for being in the study prespecified and applied uniformly to all participants? | Y | Y | Y | Y | Y | Y | Y |
| 5 | Was a sample size justification, power description, or variance and effect estimates provided? | N | Y | Y | Y | N | Y | Y |
| 6 | For the analyses in this paper, were the exposure(s) of interest measured prior to the outcome(s) being measured? | N | NA | NA | N | NA | N | N |
| 7 | Was the timeframe sufficient so that one could reasonably expect to see an association between exposure and outcome if it existed? | Y | NA | NA | Y | NA | Y | Y |
| 8 | For exposures that can vary in amount or level, did the study examine different levels of the exposure as related to the outcome (e.g., categories of exposure, or exposure measured as continuous variable)? | N | Y | Y | Y | Y | Y | N |
| 9 | Were the exposure measures (independent variables) clearly defined, valid, reliable, and implemented consistently across all study participants? | Y | Y | Y | Y | Y | Y | Y |
| 10 | Was the exposure(s) assessed more than once over time? | N | NA | N | N | NA | N | N |
| 11 | Were the outcome measures (dependent variables) clearly defined, valid, reliable, and implemented consistently across all study participants? | Y | Y | Y | Y | Y | Y | Y |
| 12 | Were the outcome assessors blinded to the exposure status of participants? | N | N | N | N | N | N | N |
| 13 | Was loss to follow-up after baseline 20% or less? | N | NA | NR | NR | NA | NR | NR |
| 14 | Were key potential confounding variables measured and adjusted statistically for their impact on the relationship between exposure(s) and outcome(s)? | Y | N | N | Y | N | N | Y |

| Quality Assessment Tool for Observational Cohort and Cross-Sectional Studies | | Ibiebele et al. (2000) | Jarallah al. (2019) | Jatrana et al. (2014) | Jiang et al. (2017) | Jin et al. (2017) | Jin et al. (2017) | Joshi et al. (2017) |
| --- | --- | --- | --- | --- | --- | --- | --- | --- |
| 1 | Was the research question or objective in this paper clearly stated? | Y | Y | Y | Y | Y | Y | Y |
| 2 | Was the study population clearly specified and defined? | N | N | N | N | Y | N | Y |
| 3 | Was the participation rate of eligible persons at least 50%? | Y | Y | NR | Y | N | N | NR |
| 4 | Were all the subjects selected or recruited from the same or similar populations (including the same time period)? Were inclusion and exclusion criteria for being in the study prespecified and applied uniformly to all participants? | Y | Y | Y | Y | Y | Y | Y |
| 5 | Was a sample size justification, power description, or variance and effect estimates provided? | Y | Y | Y | N | Y | Y | N |
| 6 | For the analyses in this paper, were the exposure(s) of interest measured prior to the outcome(s) being measured? | NA | N | N | N | N | N | N |
| 7 | Was the timeframe sufficient so that one could reasonably expect to see an association between exposure and outcome if it existed? | NA | Y | Y | NA | Y | Y | Y |
| 8 | For exposures that can vary in amount or level, did the study examine different levels of the exposure as related to the outcome (e.g., categories of exposure, or exposure measured as continuous variable)? | N | Y | N | N | Y | N | Y |
| 9 | Were the exposure measures (independent variables) clearly defined, valid, reliable, and implemented consistently across all study participants? | Y | Y | Y | Y | Y | Y | Y |
| 10 | Was the exposure(s) assessed more than once over time? | NA | N | N | NA | N | N | N |
| 11 | Were the outcome measures (dependent variables) clearly defined, valid, reliable, and implemented consistently across all study participants? | Y | Y | Y | Y | Y | Y | Y |
| 12 | Were the outcome assessors blinded to the exposure status of participants? | N | N | N | N | N | N | N |
| 13 | Was loss to follow-up after baseline 20% or less? | NA | N | NR | NA | N | N | NR |
| 14 | Were key potential confounding variables measured and adjusted statistically for their impact on the relationship between exposure(s) and outcome(s)? | Y | Y | Y | Y | Y | Y | N |

| Quality Assessment Tool for Observational Cohort and Cross-Sectional Studies | | Kang et al. (2020) | Kartal al. (2019) | Kiropoulos et al. (2011) | Kiropoulos et al. (2004) | Liddell et al. (2013) | Lies et al. (2019) | Lin et al. (2016) |
| --- | --- | --- | --- | --- | --- | --- | --- | --- |
| 1 | Was the research question or objective in this paper clearly stated? | Y | Y | NR | Y | N | N | Y |
| 2 | Was the study population clearly specified and defined? | Y | Y | NR | Y | Y | N | NA |
| 3 | Was the participation rate of eligible persons at least 50%? | Y | N | NR | Y | Y | N | Y |
| 4 | Were all the subjects selected or recruited from the same or similar populations (including the same time period)? Were inclusion and exclusion criteria for being in the study prespecified and applied uniformly to all participants? | Y | Y | NR | Y | Y | NA | NA |
| 5 | Was a sample size justification, power description, or variance and effect estimates provided? | Y | N | Y | Y | N | NA | NA |
| 6 | For the analyses in this paper, were the exposure(s) of interest measured prior to the outcome(s) being measured? | Y | N | NR | Y | Y | N | Y |
| 7 | Was the timeframe sufficient so that one could reasonably expect to see an association between exposure and outcome if it existed? | Y | N | NR | Y | N | NA | NA |
| 8 | For exposures that can vary in amount or level, did the study examine different levels of the exposure as related to the outcome (e.g., categories of exposure, or exposure measured as continuous variable)? | Y | Y | NR | Y | N | N | Y |
| 9 | Were the exposure measures (independent variables) clearly defined, valid, reliable, and implemented consistently across all study participants? | Y | Y | NR | Y | Y | N | NA |
| 10 | Was the exposure(s) assessed more than once over time? | Y | N | NR | Y | Y | N | Y |
| 11 | Were the outcome measures (dependent variables) clearly defined, valid, reliable, and implemented consistently across all study participants? | Y | Y | NR | Y | Y | NA | NA |
| 12 | Were the outcome assessors blinded to the exposure status of participants? | Y | N | Y | Y | N | NA | NA |
| 13 | Was loss to follow-up after baseline 20% or less? | Y | N | NR | Y | Y | N | Y |
| 14 | Were key potential confounding variables measured and adjusted statistically for their impact on the relationship between exposure(s) and outcome(s)? | Y | N | NR | Y | N | NA | NA |

| Quality Assessment Tool for Observational Cohort and Cross-Sectional Studies | | Lumley et al. (2018) | Maldari al. (2019) | Maneze et al. (2018) | May et al. (2014) | Meng et al. (2014) | Menigoz et al. (2016) | Momartin et al. (2004) |
| --- | --- | --- | --- | --- | --- | --- | --- | --- |
| 1 | Was the research question or objective in this paper clearly stated? | Y | Y | Y | Y | Y | Y | Y |
| 2 | Was the study population clearly specified and defined? | Y | N | Y | N | N | N | N |
| 3 | Was the participation rate of eligible persons at least 50%? | Y | NR | NR | NR | NR | NR | Y |
| 4 | Were all the subjects selected or recruited from the same or similar populations (including the same time period)? Were inclusion and exclusion criteria for being in the study prespecified and applied uniformly to all participants? | Y | Y | Y | Y | Y | Y | Y |
| 5 | Was a sample size justification, power description, or variance and effect estimates provided? | Y | N | N | Y | Y | N | N |
| 6 | For the analyses in this paper, were the exposure(s) of interest measured prior to the outcome(s) being measured? | NA | NA | NA | NA | NA | N | NA |
| 7 | Was the timeframe sufficient so that one could reasonably expect to see an association between exposure and outcome if it existed? | NA | NA | NA | NA | NA | Y | NA |
| 8 | For exposures that can vary in amount or level, did the study examine different levels of the exposure as related to the outcome (e.g., categories of exposure, or exposure measured as continuous variable)? | N | N | N | N | Y | Y | N |
| 9 | Were the exposure measures (independent variables) clearly defined, valid, reliable, and implemented consistently across all study participants? | Y | Y | Y | Y | Y | Y | Y |
| 10 | Was the exposure(s) assessed more than once over time? | NA | NA | NA | NA | NA | N | NA |
| 11 | Were the outcome measures (dependent variables) clearly defined, valid, reliable, and implemented consistently across all study participants? | Y | Y | Y | Y | Y | Y | Y |
| 12 | Were the outcome assessors blinded to the exposure status of participants? | N | N | N | N | N | N | N |
| 13 | Was loss to follow-up after baseline 20% or less? | NA | NA | NA | NA | NA | NR | NA |
| 14 | Were key potential confounding variables measured and adjusted statistically for their impact on the relationship between exposure(s) and outcome(s)? | N | N | N | N | N | Y | N |

| Quality Assessment Tool for Observational Cohort and Cross-Sectional Studies | | Nickerson et al. (2018) | Oei al. (2015) | Pasupuleti et al. (2016) | Perusco et al. (2010) | Ponsford et al. (2020) | Renzaho et al. (2014) | Renzaho et al. (2011) |
| --- | --- | --- | --- | --- | --- | --- | --- | --- |
| 1 | Was the research question or objective in this paper clearly stated? | Y | Y | Y | Y | Y | Y | Y |
| 2 | Was the study population clearly specified and defined? | N | N | N | N | N | N | N |
| 3 | Was the participation rate of eligible persons at least 50%? | Y | Y | NR | Y | N | N | NR |
| 4 | Were all the subjects selected or recruited from the same or similar populations (including the same time period)? Were inclusion and exclusion criteria for being in the study prespecified and applied uniformly to all participants? | Y | Y | Y | Y | Y | Y | Y |
| 5 | Was a sample size justification, power description, or variance and effect estimates provided? | Y | Y | Y | Y | Y | Y | N |
| 6 | For the analyses in this paper, were the exposure(s) of interest measured prior to the outcome(s) being measured? | N | NA | Y | NA | Y | NA | NA |
| 7 | Was the timeframe sufficient so that one could reasonably expect to see an association between exposure and outcome if it existed? | Y | NA | Y | NA | Y | NA | NA |
| 8 | For exposures that can vary in amount or level, did the study examine different levels of the exposure as related to the outcome (e.g., categories of exposure, or exposure measured as continuous variable)? | N | Y | N | N | N | N | Y |
| 9 | Were the exposure measures (independent variables) clearly defined, valid, reliable, and implemented consistently across all study participants? | Y | Y | Y | Y | Y | Y | Y |
| 10 | Was the exposure(s) assessed more than once over time? | N | NA | N | NA | Y | NA | NA |
| 11 | Were the outcome measures (dependent variables) clearly defined, valid, reliable, and implemented consistently across all study participants? | Y | Y | Y | Y | Y | Y | Y |
| 12 | Were the outcome assessors blinded to the exposure status of participants? | N | N | N | N | N | N | N |
| 13 | Was loss to follow-up after baseline 20% or less? | NR | NA | NR | NA | NR | NA | NA |
| 14 | Were key potential confounding variables measured and adjusted statistically for their impact on the relationship between exposure(s) and outcome(s)? | N | N | N | Y | N | Y | N |

| Quality Assessment Tool for Observational Cohort and Cross-Sectional Studies | | Rowe et al. (2020) | Sahle al. (2020) | Saltapidas et al. (2007) | Sanchez et al. (2020) | Sarich et al. (2015) | Schweitzer et al. (2011) | Schweitzer et al. (2018) |
| --- | --- | --- | --- | --- | --- | --- | --- | --- |
| 1 | Was the research question or objective in this paper clearly stated? | Y | Y | Y | Y | Y | Y | Y |
| 2 | Was the study population clearly specified and defined? | Y | Y | N | N | N | N | N |
| 3 | Was the participation rate of eligible persons at least 50%? | N | Y | NR | NR | Y | NR | NR |
| 4 | Were all the subjects selected or recruited from the same or similar populations (including the same time period)? Were inclusion and exclusion criteria for being in the study prespecified and applied uniformly to all participants? | Y | Y | Y | Y | Y | Y | Y |
| 5 | Was a sample size justification, power description, or variance and effect estimates provided? | N | N | N | N | N | Y | Y |
| 6 | For the analyses in this paper, were the exposure(s) of interest measured prior to the outcome(s) being measured? | NA | NA | NA | N | N | NA | NA |
| 7 | Was the timeframe sufficient so that one could reasonably expect to see an association between exposure and outcome if it existed? | NA | NA | NA | NA | Y | NA | NA |
| 8 | For exposures that can vary in amount or level, did the study examine different levels of the exposure as related to the outcome (e.g., categories of exposure, or exposure measured as continuous variable)? | Y | Y | N | N | N | N | N |
| 9 | Were the exposure measures (independent variables) clearly defined, valid, reliable, and implemented consistently across all study participants? | Y | Y | Y | Y | Y | Y | Y |
| 10 | Was the exposure(s) assessed more than once over time? | NA | NA | NA | N | N | NA | NA |
| 11 | Were the outcome measures (dependent variables) clearly defined, valid, reliable, and implemented consistently across all study participants? | Y | Y | Y | Y | Y | Y | Y |
| 12 | Were the outcome assessors blinded to the exposure status of participants? | N | N | N | N | N | N | N |
| 13 | Was loss to follow-up after baseline 20% or less? | NA | NA | NA | NA | N | NA | NA |
| 14 | Were key potential confounding variables measured and adjusted statistically for their impact on the relationship between exposure(s) and outcome(s)? | Y | Y | N | N | Y | N | N |

| Quality Assessment Tool for Observational Cohort and Cross-Sectional Studies | | Shamshirgaran et al. (2013) | Shamshirgaran al. (2015) | Shamshirgaran et al. (2019) | Silove et al. (2010) | Silove et al. (2007) | Slewa-Younan et al. (2020) | Slewa-Younan et al. (2014) |
| --- | --- | --- | --- | --- | --- | --- | --- | --- |
| 1 | Was the research question or objective in this paper clearly stated? | Y | Y | Y | Y | Y | Y | Y |
| 2 | Was the study population clearly specified and defined? | N | N | N | N | Y | Y | Y |
| 3 | Was the participation rate of eligible persons at least 50%? | NR | N | NR | Y | NR | NR | NR |
| 4 | Were all the subjects selected or recruited from the same or similar populations (including the same time period)? Were inclusion and exclusion criteria for being in the study prespecified and applied uniformly to all participants? | Y | Y | Y | Y | Y | Y | Y |
| 5 | Was a sample size justification, power description, or variance and effect estimates provided? | Y | N | N | Y | Y | Y | N |
| 6 | For the analyses in this paper, were the exposure(s) of interest measured prior to the outcome(s) being measured? | N | N | N | NA | NA | NA | NA |
| 7 | Was the timeframe sufficient so that one could reasonably expect to see an association between exposure and outcome if it existed? | Y | Y | Y | NA | NA | NA | NA |
| 8 | For exposures that can vary in amount or level, did the study examine different levels of the exposure as related to the outcome (e.g., categories of exposure, or exposure measured as continuous variable)? | N | N | N | N | N | N | N |
| 9 | Were the exposure measures (independent variables) clearly defined, valid, reliable, and implemented consistently across all study participants? | Y | Y | Y | Y | Y | Y | Y |
| 10 | Was the exposure(s) assessed more than once over time? | N | N | N | NA | NA | NA | NA |
| 11 | Were the outcome measures (dependent variables) clearly defined, valid, reliable, and implemented consistently across all study participants? | Y | Y | Y | Y | Y | Y | Y |
| 12 | Were the outcome assessors blinded to the exposure status of participants? | N | N | N | N | N | N | N |
| 13 | Was loss to follow-up after baseline 20% or less? | NR | NR | NR | NA | NA | NA | NA |
| 14 | Were key potential confounding variables measured and adjusted statistically for their impact on the relationship between exposure(s) and outcome(s)? | Y | Y | Y | N | Y | N | N |

| Quality Assessment Tool for Observational Cohort and Cross-Sectional Studies | | Slewa‑Younan et al. (2017) | Stanaway al. (2020) | Stanaway et al. (2011) | Steel et al. (2004) | Straiton et al. (2014) | Tan et al. (2013) | Tang et al. (2009) |
| --- | --- | --- | --- | --- | --- | --- | --- | --- |
| 1 | Was the research question or objective in this paper clearly stated? | Y | Y | Y | Y | Y | Y | Y |
| 2 | Was the study population clearly specified and defined? | Y | Y | Y | Y | Y | Y | Y |
| 3 | Was the participation rate of eligible persons at least 50%? | NR | Y | N | Y | N | NR | NR |
| 4 | Were all the subjects selected or recruited from the same or similar populations (including the same time period)? Were inclusion and exclusion criteria for being in the study prespecified and applied uniformly to all participants? | Y | Y | Y | Y | Y | Y | Y |
| 5 | Was a sample size justification, power description, or variance and effect estimates provided? | N | N | N | N | N | N | N |
| 6 | For the analyses in this paper, were the exposure(s) of interest measured prior to the outcome(s) being measured? | NA | N | N | NA | N | N | NA |
| 7 | Was the timeframe sufficient so that one could reasonably expect to see an association between exposure and outcome if it existed? | NA | Y | Y | NA | N | N | NA |
| 8 | For exposures that can vary in amount or level, did the study examine different levels of the exposure as related to the outcome (e.g., categories of exposure, or exposure measured as continuous variable)? | N | N | N | N | N | N | N |
| 9 | Were the exposure measures (independent variables) clearly defined, valid, reliable, and implemented consistently across all study participants? | Y | Y | Y | Y | Y | Y | Y |
| 10 | Was the exposure(s) assessed more than once over time? | NA | N | NA | NA | N | N | NA |
| 11 | Were the outcome measures (dependent variables) clearly defined, valid, reliable, and implemented consistently across all study participants? | Y | Y | Y | Y | Y | Y | Y |
| 12 | Were the outcome assessors blinded to the exposure status of participants? | N | N | N | N | N | N | N |
| 13 | Was loss to follow-up after baseline 20% or less? | NA | NR | NA | NA | N | NR | NA |
| 14 | Were key potential confounding variables measured and adjusted statistically for their impact on the relationship between exposure(s) and outcome(s)? | N | Y | Y | Y | Y | Y | N |

| Quality Assessment Tool for Observational Cohort and Cross-Sectional Studies | | Taylor et al. (2018) | Tran al. (2015) | Tran et al. (2014) | Vromans et al. (2020) | Weber et al. (2010) | Wu et al. (2020) | Wyk et al. (2012) |
| --- | --- | --- | --- | --- | --- | --- | --- | --- |
| 1 | Was the research question or objective in this paper clearly stated? | Y | Y | Y | Y | Y | Y | Y |
| 2 | Was the study population clearly specified and defined? | Y | Y | Y | Y | Y | Y | Y |
| 3 | Was the participation rate of eligible persons at least 50%? | Y | N | N | NR | Y | NR | NR |
| 4 | Were all the subjects selected or recruited from the same or similar populations (including the same time period)? Were inclusion and exclusion criteria for being in the study prespecified and applied uniformly to all participants? | Y | Y | Y | Y | Y | Y | Y |
| 5 | Was a sample size justification, power description, or variance and effect estimates provided? | N | N | Y | N | N | Y | Y |
| 6 | For the analyses in this paper, were the exposure(s) of interest measured prior to the outcome(s) being measured? | NA | N | N | N | N | N | NA |
| 7 | Was the timeframe sufficient so that one could reasonably expect to see an association between exposure and outcome if it existed? | NA | Y | Y | N | N | Y | NA |
| 8 | For exposures that can vary in amount or level, did the study examine different levels of the exposure as related to the outcome (e.g., categories of exposure, or exposure measured as continuous variable)? | N | Y | Y | N | N | N | N |
| 9 | Were the exposure measures (independent variables) clearly defined, valid, reliable, and implemented consistently across all study participants? | Y | Y | Y | Y | Y | Y | Y |
| 10 | Was the exposure(s) assessed more than once over time? | NA | N | N | N | N | N | NA |
| 11 | Were the outcome measures (dependent variables) clearly defined, valid, reliable, and implemented consistently across all study participants? | Y | Y | Y | Y | Y | Y | Y |
| 12 | Were the outcome assessors blinded to the exposure status of participants? | N | N | N | N | N | N | N |
| 13 | Was loss to follow-up after baseline 20% or less? | NA | N | N | N | NR | N | NA |
| 14 | Were key potential confounding variables measured and adjusted statistically for their impact on the relationship between exposure(s) and outcome(s)? | Y | Y | Y | N | Y | Y | N |

**Key:** Y = Yes; N = No; CD = cannot determine; NA = not applicable; NR = not reported
